# Decoding the prognostic landscape of LUAD: the interplay between N^6^-methyladenosine modification and immune microenvironment

**DOI:** 10.3389/fimmu.2024.1514497

**Published:** 2024-12-10

**Authors:** Quan Chen, Weijun Wan, Qing Zhao, Juan Li, Yanli Xiong, Yuchuan Yuan, Lu Tang, Xiaofeng Wu, Wei Xing, Wei Guo, Di Lu, Luoquan Ao, Xiang Xu, Xiang Ao

**Affiliations:** ^1^ State Key Laboratory of Trauma and Chemical Poisoning, Department of Stem Cell and Regenerative Medicine, Daping Hospital, Army Medical University, Chongqing, China; ^2^ Yunnan Key Laboratory of Stem Cell and Regenerative Medicine, Science and Technology Achievement Incubation Center, Kunming Medical University, Kunming, China; ^3^ Cancer Center, Daping Hospital, Army Medical University, Chongqing, China; ^4^ Department of Biochemistry and Molecular Biology, College of Basic Medical Sciences, Army Medical University, Chongqing, China; ^5^ Department of orthopedics, 953 Hospital of PLA Army, Shigatse Branch of Xinqiao Hospital, Army Medical University, Shigatse, China; ^6^ Institute of Cancer, Xinqiao Hospital, Army Medical University, Chongqing, China

**Keywords:** lung adenocarcinoma, N^6^-methyladenosine modification, tumor immune microenvironment, prognosis, predictive model, multi-omics validation

## Abstract

**Background:**

To determine the role of N^6^-methyladenosine (m^6^A) modification in the tumor immune microenvironment (TIME), as well as their association with lung adenocarcinoma (LUAD).

**Methods:**

Consensus clustering was performed to identify the subgroups with distinct immune or m^6^A modification patterns using profiles from TCGA. A risk score model was constructed using least absolute shrinkage and selection operator regression and validated in two independent cohorts and LUAD tissue microarrays. For experimental validation, the regulation of METTL3/m^6^A axis in the expression of candidate genes by RIP-qPCR assay in A549 and H460 cell lines. Co-culture experiments with human T cells were performed to evaluate the impact of METTL3 on the enhancement of anti-tumor immunity through *in vitro* experiments.

**Results:**

We identified 282 m^6^A regulator genes and 955 immune-related genes, selecting seven key genes (SFTPC, CYP24A1, KRT6A, PTTG1, S100P, FAM83A, and ANLN) to develop a risk score model using Lasso regression. High-risk patients, determined by this model, exhibited poorer prognosis, increased immune infiltration, higher tumor mutational burden, more neoantigens, and elevated PD-L1 expression. These findings were validated by two independent databases and LUAD tissue microarrays. METTL3 was found to impact the mRNA expression of these genes, with METTL3 deficiency abolishing these interactions. Inhibition of METTL3 enhanced anti-tumor immunity, T cell activation, exhaustion, and infiltration *in vitro*.

**Conclusion:**

This risk score system shows promise for prognostic prediction and the development of personalized treatment strategies for LUAD patients.

## Introduction

Lung adenocarcinoma (LUAD) is the most prevalent form of lung carcinoma that has an average 5-year survival rate of 20% (1, 2). As an immune-sensitive malignancy, the tumor microenvironment of LUAD is characterized by multiple types of immune cell infiltration (3). Contrary to the anticipated outcome of the immune system to identify and diminish cancer cells owing to their distinct, and often extensive, mutational characteristics, tolerance serves as the natural equilibrium between the immune system and cancer. Various mechanisms involving regulatory immune cells, immunosuppressive cytokines, and chemokines work together to maintain the tolerance (4). Considering this, the use of monoclonal antibodies that block these pathways has emerged as a potent tool in oncology. Recently, the application of immune checkpoint inhibitors (ICIs) has yielded impressive outcomes for patients with LUAD (5, 6). However, immunotherapy has demonstrated beneficial outcomes in fewer than 20% of patients diagnosed with LUAD. Recent clinical studies have shown that the absence of specific biomarkers that correlate with prognosis and ICIs response is primarily why approximately half the patients do not show clinical or survival improvements (7). Therefore, exploring for biomarkers is essential to identifying the patients who will be suitable for monotherapy, as well as to provide timely indications of treatment response, drawing upon our evolving scientific understanding of the biological mechanisms underlying immune pathway inhibition.

The N^6^-methylandenosine (m^6^A) modification, which is present in all eukaryotic RNA molecules, is regulated by some signals, including methyltransferases, signal transducers, and demethylases (8). Current findings have highlighted the significant role of m^6^A modifications in cancer biology, particularly in tumor progression and response to therapy (9, 10). Previous research has revealed that m^6^A methyltransferase enhances PD-L1 expression after transcription, which indicating the modification is crucial for the regulation of certain immunological characteristics in the tumor immune microenvironment (TIME) (11). The signal transducer deficiency improves the efficacy of anti-PD-1 treatment via the m^6^A–p65–CXCL axis (12). Therefore, understanding the link between m^6^A modification regulators and genes encoding immunological functions is essential for optimizing cancer treatment outcomes. In accordance with the m^6^A regulators, some researchers have developed predictive models for the survival outcomes of LUAD patients and their association with immune checkpoint inhibition (13, 14). An m^6^A-based scoring system has been developed to differentiate patients who displayed increased infiltration of CD8+ T cells and exhibited heightened sensitivity to immunotherapy (15). However, no experimental or real-world validation has been conducted and some models focus solely on genetic or transcriptomic data without integrating comprehensive immune profiling, which is essential for understanding the tumor-immune interplay.

In this study, we conducted a prognostic evaluation for both m^6^A regulatory genes and immune-associated genes in LUAD, and selected seven signatures to develop a scoring system by assessing risk profiles. Furthermore, we investigated the association among risk ratings, tumor immunity status, and m^6^A regulators. The model was validated using seven LUAD tissue microarrays, confirming its robustness and applicability in real-world settings. Experimental validation showed that the expression of these candidate genes was directly regulated through an m^6^A-dependent mechanism, and inhibition of METTL3 enhances anti-tumor immunity, T cell activation, exhaustion, and infiltration *in vitro*. This study highlights the potential of the risk score system for prognostic prediction and the development of more effective personalized treatment strategies for LUAD patients.

## Materials and methods

### Sources and preprocessing of data

This study was conducted using The Cancer Genome Atlas (TCGA, https://cancergenome.nih.gov/) and Gene Expression Omnibus (GEO, National Center for Biotechnology Information, USA)) databases. We acquired transcript sequencing array data as the training cohort, measured in fragments per kilobase million (FPKM), for individuals identified as LUAD from TCGA. Additionally, we downloaded the transcriptome profile expression levels of two cohorts (GEO: GSE30219 and GSE50081) for external validation. The cohorts selected for analysis were based on the following criteria: 1. large-scale human samples of mRNA gene-expression patterns from untreated primary LUAD tissues with more than 30 samples; 2. assessed on the same technological platform that stores raw expression data and clinical information (such as survival times, censored information, and TNM stage); and 3. Peer reviews or publications in scientific journals proved or evaluated the data quality. A log2 scale was applied to all raw data after quantile standardization. Subsequently, we exercised prudence by excluding genes from the dataset with expression levels of 0 FPKM in at least 50% of the samples.

### Cell culture and transfection with small interfering RNA

The human non–small-cell lung cancer (NSCLC) cell line (A549 and H460) was acquired from the American Type Culture Collection (ATCC, USA). The DMEM medium (BI, Israel) supplemented with 10% fetal bovine serum (BI, Israel) was used to cultivate the above cells in a temperature of 37°C under 5% CO2. To knock down METTL3, METTL14, and WTAP, all cells were transfected with small interfering RNA (siRNA) targeting these genes or control siRNA using Lipofectamine RNAiMAX (Invitrogen, USA) as per the manufacturer’s instructions. Untreated cells served as negative controls, and siRNA-targeting scrambled sequences were used as a transfection control. Transfection efficiency was validated using RT-qPCR, confirming significant knockdown of METTL3, METTL14, and WTAP, as shown in **Supplementary Figure 4**. **Supplementary Table 1** listed these siRNA sequences.

### Generation of the stable cell lines

For METTL3 knockdown, lentiviral vectors harboring shRNA for knockdown and overexpression of METTL3 and negative control underwent syncretization and then cloned into pLKO.1 vector. The plasmids were transfected using lipofectamine LTX and Plus™ Reagent (Invitrogen, USA) into A549 cells according to the manufacturer’s protocol. The sequences are presented in **Supplementary Table 1**. Briefly, stably transfected cells were selected with 10 μg/ml puromycin (MCE, USA) for 3 weeks.

### Western blot analysis

The proteins of cells were extracted using the RIPA buffer, which was cooled before use (Beyotime, China). Identical protein samples were measured, loaded, separated on a 10% SDS-PAGE, and then transferred to 0.45 μm PVDF membranes (Beyotime, China). Following a 1.5-hour blocking step with 5% non-fat milk in TBST, the membranes were subjected to overnight incubation at 4°C with the primary antibodies (**Supplementary Table 2**). Later on, the secondary antibodies were introduced and the concoction was subjected to incubation at room temperature for one hour. The proteins in the immunoblots were identified using the GelDoc XR System (BioRad, SA).

### Co−culture experiments

Human T cells were isolated from three NSCLC patients. After obtaining informed consent, 20 ml of peripheral venous blood was collected from the donor and T cells were then isolated via density gradient centrifugation using Ficoll-Paque solution (Biolegend, USA). The isolated T cells were subsequently cultured in Roswell Park Memorial Institute (RPMI)-1640 medium (BioInd, Israel), supplemented with 10% fetal bovine serum (Gibco, USA) and IL-2 (200 U/mL; SinoBiological, China). PBMCs were added to the A549 cell cultures at a ratio of 1:5 (A549 cells: PBMCs) in fresh complete medium, with or without 10 μg/ml atezolizumab (Genentech, USA), and incubated for 48 hours at 37°C in a humidified atmosphere containing 5% CO2. The supernatant was isolated from each group and the LDH release assay (Beyotime, China) was performed based on the instructions from the manufacturer. Absorbance was detected at 490 nm by Biotek microplate reader. In addition, the supernatant was also used for the enzyme-linked immune-sorbent assay (ELISA) for quantifying IFN-γ and IL-2 production (R&D System, USA) as per instructions given by the manufacturer. Processed data from plate readings taken at 450 nm.

### LUAD tissue sample and immunochemistry

Commercially available tissue microarray slides (HLugA180Su07) containing 93 histologically confirmed LUAD tissues were purchased from Biochip (Shanghai Biochip Co., Ltd., China) for immunohistochemistry (IHC) analysis. Immunohistochemical staining was performed on tissue microarrays (TMAs) incubating with the specific antibodies (**Supplementary Table 2**) overnight at a temperature of 4°C. Subsequently, they were incubated with polyclonal peroxidase-conjugated anti-rabbit IgG (Boster Biological Technology co.ltd, USA) at room temperature for 20 min, as per the instructions provided by the manufacturer. Three expert pathologists blind to the clinical information independently graded each tissue sample. The intensity of staining was categorized as follows: 0 (negative), 1(weak), 2 (moderate), or 3 (strong). The extent of staining varied according to the proportion of positive cells (out of 200 cells examination): 0 (less than 5%), 1 (5%–25%), 2 (26%–50%), 3 (51%–75%), or 4 (>75%). The IHC expression scores were determined by multiplying the staining intensity by the staining extent, and then the scores were normalized by the z-score in order to calculate the risk scores.

### RT-qPCR

RNA was isolated from the cells using the TRIzol Reagent (Invitrogen, USA) according to the manufacturer’s instructions. Reverse transcription of the isolated RNA was performed using HiScript II Q RT SuperMix for qPCR (+gDNA wiper) (Vazyme, China). RT-qPCR was performed using the AceQ qPCR SYBR Green Master Mix (Vazyme, China). The mRNA levels were standardized using glyceraldehyde-3-phosphate dehydrogenase (GAPDH) as the reference gene. The oligonucleotide sequences are listed in **Supplementary Table 1**.

### RNA-binding protein immunoprecipitation assay

The Magna RIP Kit (Millipore, MA, USA) was used to conduct the RIP assay. Briefly, 5 μg anti-METTL3 (Abcam, USA) or anti-m^6^A (Millipore, Germany) and anti-rabbit IgG (Millipore, Germany) were incubated with 50 μL of magnetic beads before cell lysates were added (approximately 2 × 10^7^ cells per sample). Following six rounds of washing, the RNA–protein immunoprecipitation (IP) complexes were incubated in proteinase K digestion solution to extract the proteins. Finally, the RNA was purified for RT-qPCR analysis after being extracted using phenol–chloroform. Normalizing relative enrichment to the input was done as % input =1/10 × 2^Ct [IP] – Ct [input]^.

### Functional investigation and determination of genes related to m^6^A and the immune system

To discern the relationship between m^6^A and immune status, the t-distributed Stochastic Neighbor Embedding (t-SNE) algorithm was performed (16). Moreover, signature gene sets exhibit consistent expression and serve as summaries of certain clearly defined biological states or processes. According to previous publications, we retrieved expression matrixes of m^6^A regulators (17). Additionally, 29 immune-related genes were selected, reflecting diverse array of immune cell types, roles, and pathways (IMMPORT) (18). We employed the Non-negative Matrix Factorization (NMF) technique to cluster the expression patterns of immune- or m^6^A-associated genes. We used k-means clustering because it allows for the identification of distinct subgroups based on m^6^A modification patterns, assuming that the data are relatively homogeneous within each cluster. Using Cox regression analysis, we assessed the correlation between each potential gene and overall survival (OS). Three clusters were determined to be the optimal number when the correlation coefficient decreased. Using the aforementioned immune/m^6^A gene mRNA expression data and the t-SNE algorithm, LUAD subtypes were identified. The differentially expressed genes (DEGs) were determined with a criterion of |logFC| > 0.585 and P<0.05, after controlling for false discovery rate (FDR). In addition, we performed Kyoto Encyclopedia of Genes and Genomes (KEGG) (https://www.kegg.jp/) and Gene Ontology (GO) pathway analyses (https://david.ncifcrf.gov//) using the R Cluster-Profiler. Pathways were considered significant when P and q values were below the 0.05 threshold.

### Development and validation of risk scoring system

Genes that overlapped between the m^6^A- and immune-associated DEGs were selected for further analysis. The risk scoring system was established using Cox regression analysis and the Least Absolute Shrinkage and Selection operator (LASSO) (19). Using five-fold cross-validation (CV), we were able to find the optimal parameters while reducing the bias that overfitting the training samples may induce. The prognostic roles of candidate genes were subsequently investigated using multivariate Cox regression analysis. Risk scores were generated by multiplying the multivariate Cox regression’s coefficient of gene expression. All patients were categorized as either high- or low-risk according to the median risk score. The data were analyzed using Kaplan-Meier survival analysis, and statistical significance was determined by log-rank tests. We tested the model’s prediction power using a receiver operating characteristic (ROC) curve.

### Analysis of the interrelationships between DEGs associated with immune function

To assess gene set enrichment in transcriptomes, we conducted Gene Set Variation Analysis (GSVA). This technique scores gene sets to convert gene expression levels to pathway levels, thus determining the biological function of samples (20). We evaluated the immunological state and m^6^A levels in risk groups using data from the Molecular Signature Database, with significant results at FDR q < 0.25 and P < 0.05. Immunocyte infiltration in the groups was analyzed via the CIBERSORT algorithm, focusing on 22 distinct immunocyte subunits. The TIMER (version 2.0) database provided data on tumor-infiltrating immune cell abundance (21). Spearman’s correlation analysis was employed to study the relationship between gene expression and immune cell concentrations. Additionally, using data from the Genomics of Drug Sensitivity in Cancer database (GDSC), we estimated the IC50 of chemotherapeutic medications through regression modeling.

### Statistical methods

Data analysis was carried out using the R (v.4.2.0) software. The Chi-square test was used for the analysis of qualitative variables. A statistically significant result was defined as a significance level of P < 0.05, unless specified otherwise, in specific conditions as outlined independently.

## Results

### Selection of the immune and the m^6^A-associated DEGs within LUAD

**Figure 1:**
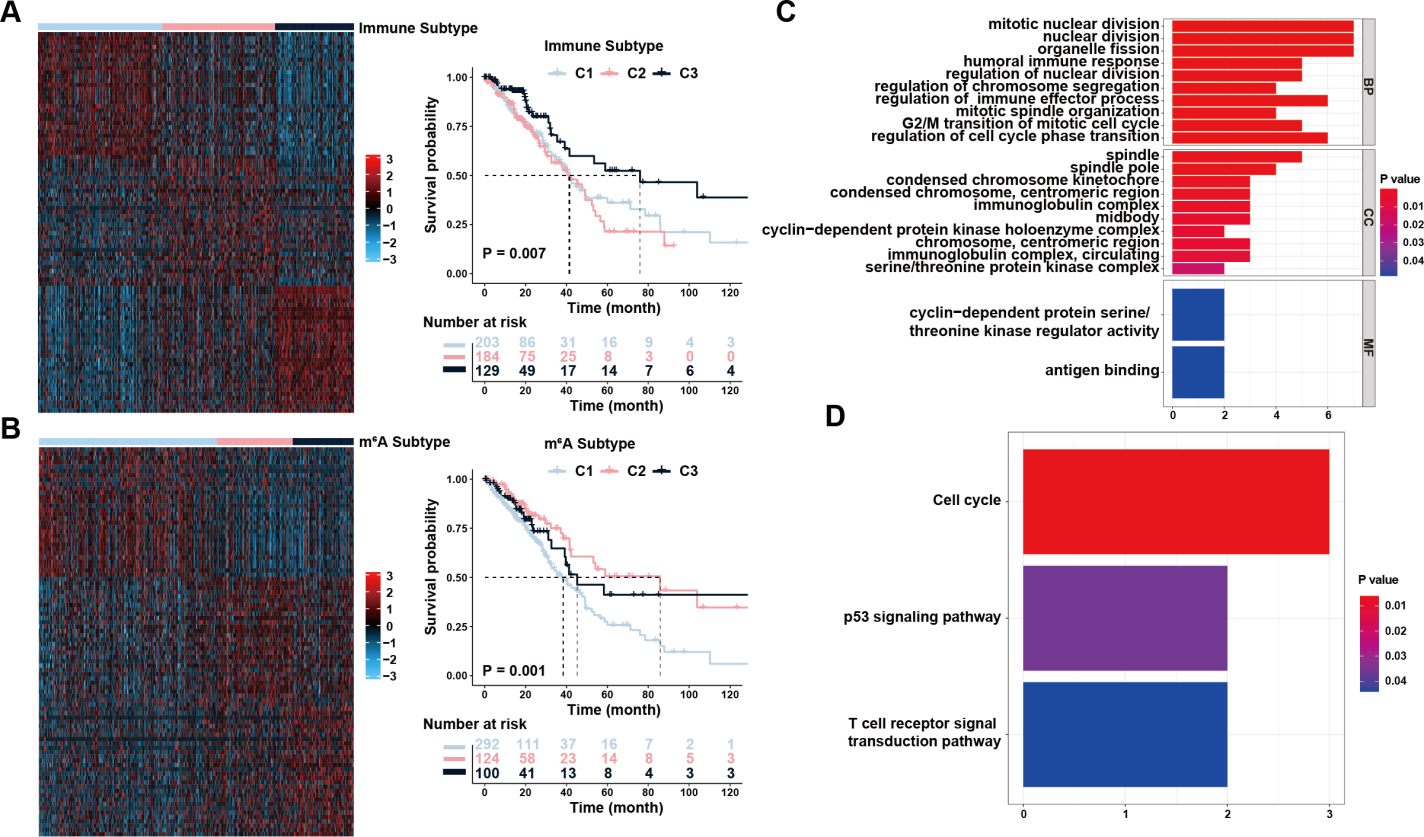
Identification of immune and m^6^A status and the differentially expressed genes (DEGs). **(A)** Unsupervised clustering of immune-related genes to classify patients into different genomic subtypes. **(B)** Unsupervised clustering of m^6^A-related genes to classify patients into different genomic subtypes. **(C, D)** Functional annotation for overlapping DEGs using GO enrichment analysis **(C)** and KEGG enrichment analysis **(D)**. The color depth of the bar plots represented the number of genes enriched.

The processed original mRNA expression data for LUAD were acquired from the TCGA database. Subsequently, 1811 immunity genes and 1670 immune regulatory factors were identified from the IMMPORT database. We further determined three immune clusters and calculated the Euclidean distance using t-SNE for each patient (**Figure 1A** and **Supplementary Figures 1A, B**). Statistically significant disparities were observed across the three clusters using survival analysis. Notably, C3 exhibited longer median survival when comparing both C1 and C2 clusters (**Figure 1A**). Similarly, three m^6^A clusters were detected based on the m^6^A regulators expression matrices. The results indicated cluster C2 had a comparatively longer median survival time (**Figure 1B** and **Supplementary Figures 1C, D**). Furthermore, we identified 955 immune-associated DEGs and 282 m^6^A -associated DEGs. Among these, 145 genes were found to be co-expressed in both the immunological and m^6^A subtypes, making them potential candidate DEGs for further investigation.

Using pathway analysis, the possible roles of the co-expressed DEGs were determined (Fisher’s exact test, P <0.05). GO enrichment analysis yielded biological process (BP), molecular function (MF), and cellular component (CC) terms. For BP, DEGs were predominantly enriched in nuclear division, regulation of cell-cycle phase transition, regulation of immune effector processes, and immune responses. In the MF, the majority of DEGs were involved in antigen binding and cyclin-dependent protein serine/threonine kinase regulatory activity. The spindle, immunoglobulin complex, and midbody were most abundant in CC enrichment (**Figure 1C**). KEGG pathway enrichment analysis showed that DEGs had a significant enrichment mostly in the Cell cycle, p53 signaling, and T cell receptor signal transduction pathways (**Figure 1D**). Collectively, the pathway enrichment results suggested that the DEGs we chose have a tight connection to cell proliferation and immune response, which may not only serve as prognostic indicators for patients with LUAD but also contribute significantly to the interplay between immune infiltration and well-established signaling pathways associated with tumor development and invasion.

### Construction and validation of the risk scoring system

**Figure 2:**
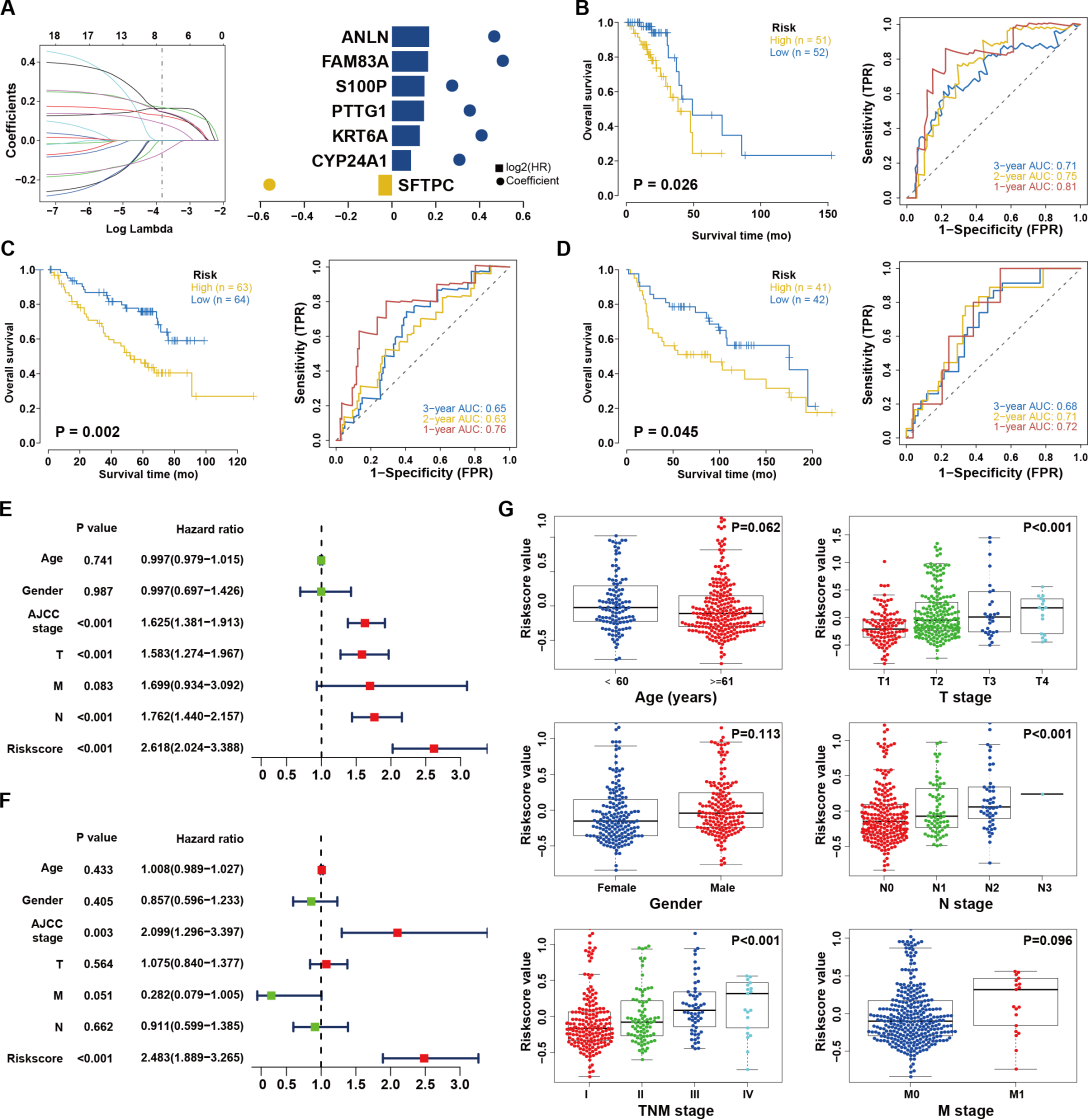
Construction and validation of the risk score system. **(A)** Least absolute1 shrinkage and selection operator (LASSO) regression was performed, calculating the minimum criteria and coefficients. **(B)** Kaplan–Meier analysis between the high-risk subgroup and low-risk subgroup in TCGA dataset. **(C)** External validation of risk score system in GEO database (GSE30219). The ROC curves predicting 1/2/3-year survival. **(D)** External validation of risk score system in GEO database (GSE50081). The ROC curves predicting 1/2/3-year survival. **(E, F)** Univariate **(E)** and multivariate **(F)** Cox analyses of clinical parameters and lasso risk for overall survival. The covariables are the N stage, T stage, AJCC (American Joint Committee on Cancer) stage, and gender of the LUAD patients. **(G)** Stratified analysis of clinical characteristics for the risk score value. The Kruskal-Wallis test was used to compare the statistical difference.

There were 18 immunologically and m^6^A-linked DEGs that contributed to OS after conducting the univariate Cox regression analysis (**Supplementary Table 3**). We then used LASSO to identify seven signatures for a risk-based prognostic assessment model (**Figure 2A** and **Supplementary Figure 2A**). We used the following formula to calculate risk scores: Risk Score = SFTPC*(-0.061883497) + CYP24A1*0.086651739 + KRT6A*0.127175139 + PTTG1*0.146161987 + S100P*0.14825993 + FAM83A*0.164912979 + ANLN*0.169266477. All patients were classified as the high-risk or low-risk cohort based on the median risk score. In both the training and internal test set, the high-risk group exhibited a lower OS compared to the OS seen in the low-risk group (P < 0.001 and P=0.026, **Supplementary Figure 2B** and **Figure 2B**) and the AUC values of the risk score for 3-year survival were 0.70 and 0.71 (**Supplementary Figure 2C** and **Figure 2B**). Subsequently, two additional independent cohorts were included for external validation (**Supplementary Tables 4**, **5**). As expected, high-risk patients in both cohorts had shorter OS compared to low-risk individuals (P = 0.002 and 0.045, respectively, **Figures 2C, D**). The AUCs with regard to 3-year OS were 0.65 and 0.68, respectively (**Figures 2C, D**). This further implied that the risk score system showed excellent repeatability and stability during validation. Furthermore, we performed Cox regression analyses and determined that the risk score served as a reliable and independent prognostic indicator (**Figures 2E, F**). Moreover, there was a substantial correlation between the risk scores and the tumor, node, metastasis (TNM), T, and N stages (**Figure 2G**). Taken together, the risk score model we constructed is not only capable in predicting the survival but also has a noteworthy connection with clinical characteristics.

### Assessment of TIME status in cohorts with varying levels of risk

**Figure 3:**
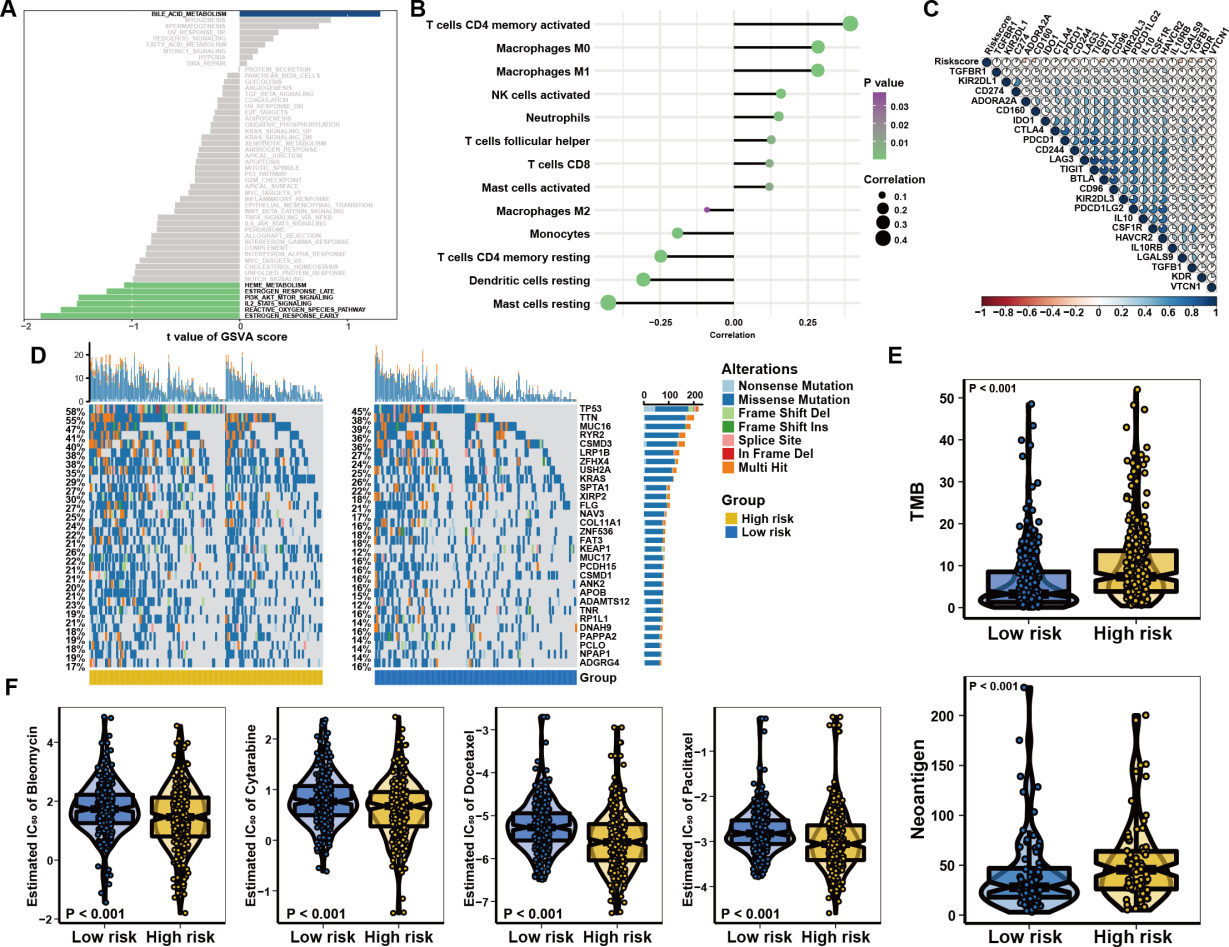
Characteristics of the risk score system in immune subtypes. **(A)** GSVA analysis revealing immune-related biological processes correlated with the signature. **(B)** Relationships between the risk model and infiltration abundances of immune cells. **(C)** Correlation between the risk score and immune checkpoint expression. **(D)** The water-fall plot of tumor somatic mutation established by those with high-risk score (left) and low-risk score (right). Each column represented individual patients. **(E)** The correlation between risk score and TMB or Neoantigen, the comparison was conducted by Wilcoxon test. **(F)** Distribution of the estimated IC50 and drug sensitivities comparison between high and low risk score groups.

To evaluate the risk cohorts and their correlation with immune status, we primarily programmed GSVA and suggested the risk cohorts were enriched in the IL2-STAT5, PI3K/AKT/MTOR, and Reactive oxygen species pathways (**Figure 3A** and **Supplementary Figures 3A, B**). Furthermore, assessment of immune cell infiltration found that the risk score was positively correlated with T cell CD4 + memory activation, Macrophages M0, Macrophages M1, activated NK cells, follicular helper T cells, CD8 + T cells, and activated Mast cells, but negatively associated with Macrophages M2, Monocytes, resting CD4 + memory T cells, dendritic cells, and resting mast cells in patients with different risk levels (**Figure 3B** and **Supplementary Figure 3C**). Interestingly, the expression of PD-L1, CTLA-4, and IDO1 increased as the risk score elevated (**Figure 3C** and **Supplementary Figure 3D**). Genetic mutation analysis revealed that individuals considered to be at high risk had higher incidences of TP53, TTN, and MUC16 mutations (**Figure 3D**). Quantitative analysis further confirmed that load of tumor mutational burden (TMB) and neoantigen levels were increased in the high-risk group (**Figure 3E**). In addition, we predicted the chemosensitivity of each tumor sample and the results indicated that low-risk individuals showed greater chemosensitivity to Bleomycin, Cytarabine, Paclitaxel and Docetaxel (**Figure 3F**). Taken together, the findings indicate that the low-risk group could exhibit increased sensitivity to conventional chemotherapy. However, patients with high-risk scores exhibited higher immune cell infiltration and were positively correlated with the expression of immune checkpoints, indicating that they may potentially receive therapeutic benefits from immunotherapy.

### Clinical experimental validation in LUAD tissue microarrays

**Figure 4:**
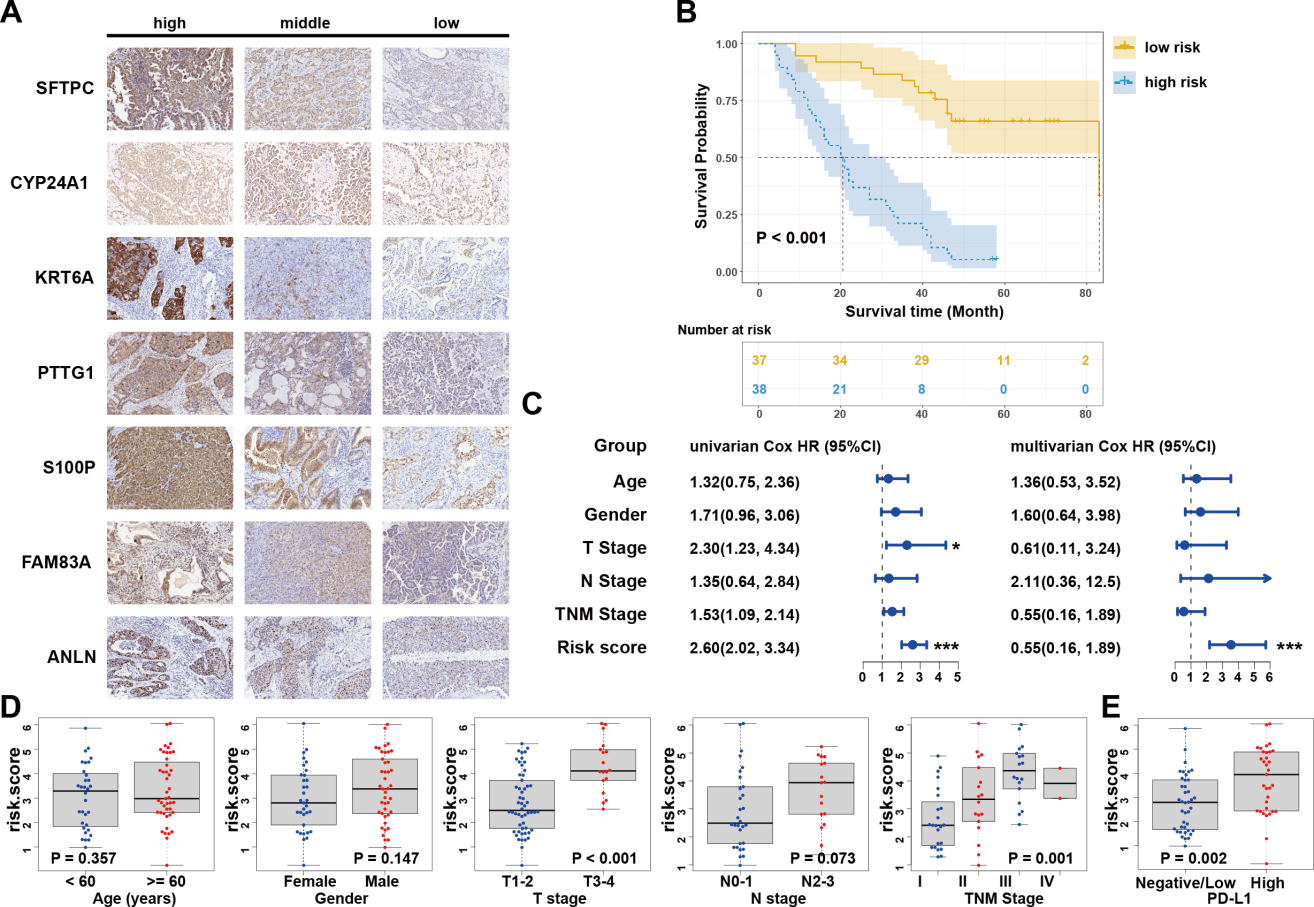
Clinical experimental validation in LUAD tissue microarrays. **(A)** IHC assay of the seven candidate signatures. **(B)** Kaplan–Meier curves for high and low risk score patient groups in LUAD tissue microarray data. **(C)** Univariate and multivariate Cox analyses of clinical parameters and lasso risk for overall survival. The covariables are the N stage, T stage, TNM stage, and gender of the LUAD patients. **(D)** Stratified analysis of clinical characteristics for the risk score value. The Kruskal-Wallis test was used to compare the statistical difference. **(E)** Correlation between risk score and PD-L1 expression in tissue microarray data. Wilcoxon test is shown in the graphs. .

Due to the proteins associated with LUAD and the TIME perform important biological functions, we conducted further experimental verification using LUAD tissue microarrays and IHC (**Figure 4A**). Following the exclusion of invalid samples, the risk score for every LUAD patient was determined using the aforementioned formula and further classified as low- or high-risk according to the median. Survival analysis revealed that patients with high risk exhibited worse OS (P<0.001; **Figure 4B**). The Cox regression analyses also showed that the risk score was the only independent prognostic indicator (**Figure 4C**). Consistent with prior findings, there was a substantial correlation between the risk scores and both TNM and T stages, which indicated a robust relationship between the risk score and tumor invasion (**Figure 4D**). Notably, PD-L1 protein expression levels were significantly higher in the high-risk group of patients (P = 0.002; **Figure 4E**). Collectively, these results experimentally verified the stability and reliability in the scoring system at protein level.

### METTL3-mediated m^6^A modifications regulate the expression of candidate genes and enhance immune responses *in vitro*

**Figure 5:**
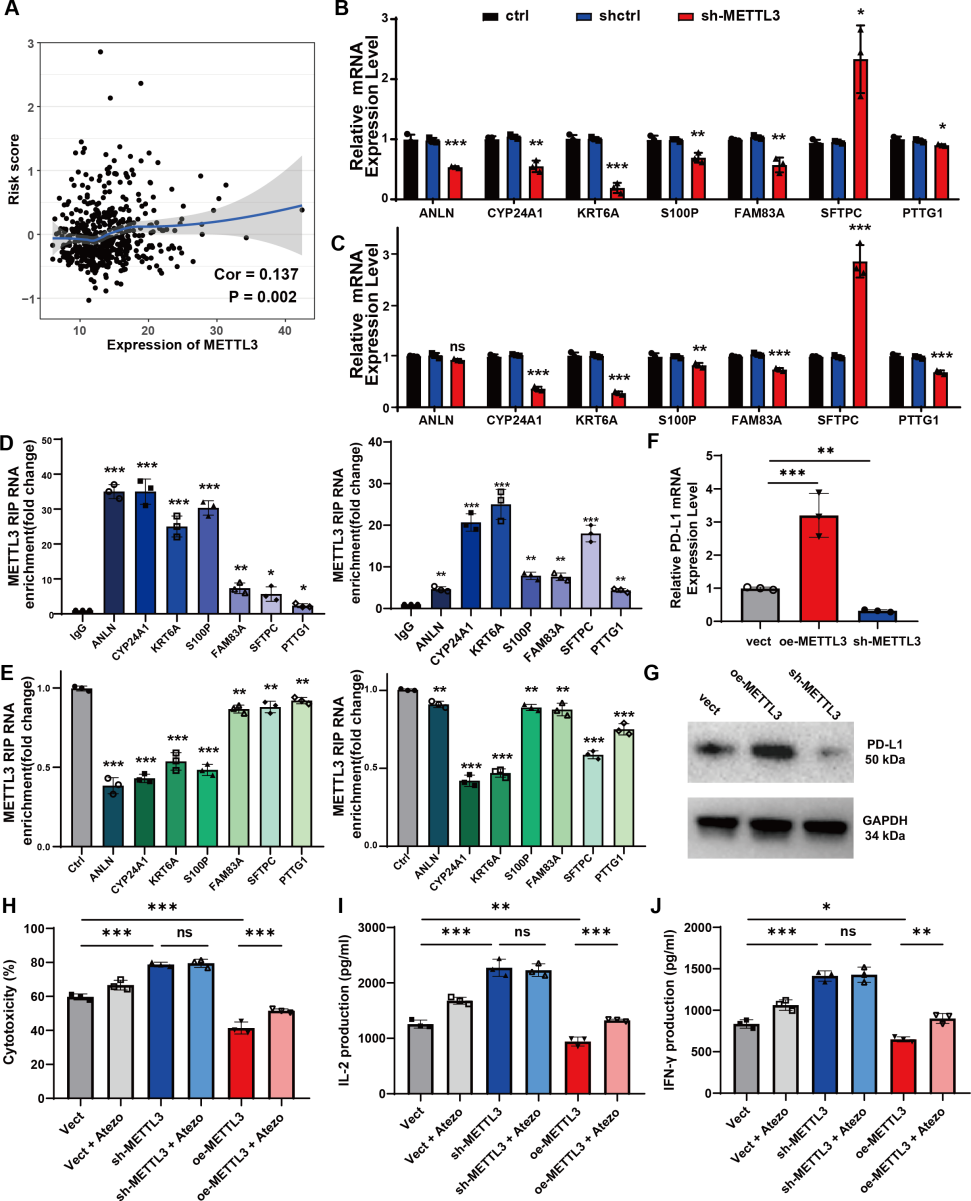
The METTL3-mediated m^6^A alteration in lung cancer regulates seven candidate signatures and enhance immune responses *in vitro*. **(A)** The correlation analysis of METTL3 expression and risk scores by Spearman test. **(B)** The relative mRNA expression of seven candidate genes after METTL3 knocking down (METTL3-KD) in A549 cells. **(C)** The relative mRNA expression of seven candidate genes after METTL3-KD in H460 cells. **(D)** Enrichment of METTL3 on mRNA compared to IgG was analyzed by RIP-qPCR assay in A549 cells (left) and H460 cells (right). **(E)** The interaction between METTL3 and mRNA in A549 cells (left) and H460 cells (right) with METTL3 knockdown. **(F)** The relative mRNA expression of PD-L1 in different groups of A549 cells with stable METTL3 overexpression (oe-METTL3) and METTL3 knockdown (sh-METTL3). **(G)** Western blotting analysis of PD-L1 in A549 cells with oe-METTL3 and sh-METTL3. **(H)** LDH release assay was used to measure cytotoxicity after 48 hours of co-culture. **(I, J)** The levels of IL-2 **(I)** and IFN-γ **(J)** in the co-culture medium was determined by ELISA. Data are represented as mean ± SEM of three independent experiments. Statistical significance was calculated by Student’s t-test. *P<0.05, **P<0.01, ***P<0.001, ns, non-significant.

As a crucial component for RNA m^6^A modification, METTL3 is essential for the regulation of TME and antitumor immunity in NSCLC (8, 22). To investigate regulatory role of METTL3 in the risk score model, we initially found a positive correlation between METTL3 expression and risk scores using Spearman correlation (R = 0.137, P<0.05; **Figure 5A**). Additionally, m^6^A modification data for candidate signatures were retrieved from the m^6^A target database (23). We subsequently investigated whether METTL3 exerts regulatory effects on candidate signatures, given its role as a key m^6^A writer in lung cancer. In A549 cells, METTL3 knockdown resulted in significant inhibition of CYP24A1, KRT6A, S100P, FAM83A, PTTG1, and ANLN expression, while SFTPC expression was significantly enhanced (**Figure 5B**). Similar expression changes were observed in H460 cells, except for ANLN, which did not show significant alteration (**Figure 5C**). This discrepancy likely reflects the distinct genetic backgrounds and m^6^A regulatory landscapes of the two cell lines, highlighting the complexity of m^6^A modifications. To confirm the role of METTL3 as the potential “writer”, we evaluated the direct binding interaction between METTL3 and the mRNAs of the seven candidate genes using RIP-qPCR assays in both cell lines. There was significant METTL3 enrichment in the mRNAs of all seven candidate genes (**Figure 5D**), and this enrichment decreased upon METTL3 silencing (**Figure 5E**). These findings confirm that METTL3 directly binds to and potentially methylates the mRNAs of the candidate genes in an m^6^A-dependent manner.

Subsequently, we explored the functional consequences of METTL3 modulation. We constructed A549 cell lines with stable overexpression (oe-METTL3) and knockdown (sh-METTL3) of METTL3 (**Supplementary Figure 4A**). The results found that oe-METTL3 increased PD-L1 expression, while sh-METTL3 decreased PD-L1 expression at both the mRNA and protein levels (**Figures 5F, G**). Coculture experiments with A549 cells and PBMCs demonstrated that METTL3 knockdown significantly enhanced T cell-mediated cytotoxicity, whereas METTL3 overexpression inhibited these T cell functions; notably, the addition of atezolizumab partially restored T cell antitumor effects with METTL3 overexpression (**Figure 5H**). Furthermore, T cell proliferation, indicated by increased IL-2 and IFN-γ production, was enhanced following METTL3 knockdown, while METTL3 overexpression inhibited these functions and atezolizumab also mitigated the inhibitory effects of METTL3 overexpression on T cell activity to some extent (**Figures 5I, J**). Collectively, these results indicate that METTL3-mediated m^6^A modifications regulate candidate gene expression in a cell line-specific manner and modulate the antitumor immune response, underscoring the potential of targeting METTL3 in NSCLC therapy.

## Discussion

Currently, enhanced understanding will facilitate the development of novel strategies to identify and eliminate high-risk groups before they develop cancerous conditions, thereby avoiding wasteful treatments for lesions with a poor likelihood of responding to immunotherapy (24, 25). In the present research, the genomic data of LUAD patients was initially integrated to comprehensively evaluate the m^6^A and TIME patterns and then collected the co-expression signatures between distinct patterns. Furthermore, we established a risk-scoring system based on seven candidate signatures (SFTPC, CYP24A1, KRT6A, PTTG1, S100P, FAM83A, and ANLN) to predict the survival benefits. Patients who were determined to be low-risk had significantly better OS by external validation. We also built a nomogram model that incorporated clinicopathological characteristics and prognostic risk ratings (**Supplementary Figure 2D**). The nomograms outperformed the other methods in predicting 5- and 7-year OS, as shown in the calibration chart (**Supplementary Figure 2E**). In addition, a nomogram that integrates risk scores and other clinical variables was constructed to provide a quantitative approach for clinical treatment. Furthermore, 93 surgical specimens were selected as independent clinical validation cohorts. A prolonged OS was also seen in those with lower scores. Additionally, risk score was the only stable and independent factor affecting survival across multiple cohorts. Taken together, we will get a better knowledge of tumor treatment by the risk scoring system established based on a large scale of LUAD cohort.

The TIME is considered a major contributor to the efficacy of both chemotherapy and immunotherapy in LUAD (26). m^6^A modification has a critical role in immune cell infiltration characterization during the TIME (11, 27, 28). In our model, the signatures that comprised the risk score model were reported to have a positive correlation with TIME. PTTG1 has been implicated in T cell cycle-dependent mechanisms and further activates T cells (29). Tumor-infiltrating immune cells are stimulated by S100P through the activation of the receptor for advanced glycation end products, and this molecule could serve as a promising biomarker for immunosuppressive microenvironment (30, 31). FAM83A, on the other hand, stimulates the expression of PD-L1 via ERK pathway and lowers immunocyte activity in LUAD (32). In agreement with these findings, the above-mentioned genes contributed to a positive index for the high-risk–scoring cohort.

We further questioned whether risk signatures play an important role in TIME. We found that TMB, neoantigens, and genetic alterations levels were increased in the high-risk group. For the analysis of the immune panorama, the risk score was found to be consistent with a higher proportion of activated CD4 + memory T cells, Macrophages M1, activated NK cells, and CD8 + T cells in the TIME. These results suggest that the TIME of high-risk patients exhibits “hot immune” conditions. They accumulate mutations that cause tumor cells create surface-bound neoantigens; this makes the tumor easier for the immune system to recognize and, hopefully, trigger a robust immune response (33, 34). However, an inverse relationship was found between the risk score and the HLA-D family, indicating the presence of a suppressive tumor immune state (35). Collectively, the high-risk LUAD patients in the present study appeared to exhibit a high percentage of immune cell infiltration microenvironment but under an immune dysfunctional condition. Researchers have mapped the type of immunocyte landscape during the TIME in advanced LUAD and similarly found a greater abundance of CD8+ T cells and macrophages infiltrating the tumor, as well as increased expression of immunosuppressive markers (36, 37), which indicates an anergic state in immune cell reactions to tumors. We gathered the expression profiles of immunosuppressive markers that have been shown to indirectly foretell the efficacy of immunotherapy. A positive association between the risk score and various immune checkpoints was shown by our data. Consistent with these results, increased TMB levels were associated with immune cell infiltration and higher expression of immunosuppressive checkpoints, resulting in increased sensitivity to ICIs in NSCLC (38). However, owing to the lack of ICI records, we preliminarily speculated the potential candidates for immunotherapy who are at high risk by comparing the relationship between risk scores and these verified biomarkers. In addition, patients with elevated risk scores exhibited reduced sensitivity to standard chemotherapeutic medicines for LUAD and were in an immunosuppressive state. Remarkably, the chemotherapy response is also affected by TIME and the activation of tumor-infiltrating immunocytes. The current clinical trials have demonstrated that combining with immunotherapy shows more effectiveness than chemotherapy alone, as well as any other combination of immunotherapy or single-agent therapy (39, 40). Therefore, we suggest prioritizing the administration of both chemotherapy and immunotherapy for patients with LUAD identified as high-risk in our approach.

In this study, the correlation between METTL3 expression and lung cancer cells was investigated *in vitro*. These results suggested that after knocking down METTL3 in LUAD cells (both A549 and H460 cell lines), the seven candidate genes were directly regulated in the same manner as in the risk score system. In addition, RIP analysis revealed enrichment of METTL3 with the mRNAs of these molecules, and this connection was disrupted by METTL3 deficiency. In addition to its role in gene expression, METTL3 plays a crucial part in immune regulation, particularly in mediating the mechanisms of immune regulatory signaling molecules. Specifically, METTL3 enhances the immunosuppressive capacity of myeloid cells that infiltrate tumors (41). Suppression of METTL3 has been shown to reduce the immunosuppressive environment, thereby enhancing immune surveillance. Notably, combining METTL3 suppression with anti-PD1 treatment has demonstrated promising effectiveness against tumors (42–44). Based on m^6^A regulators, the scoring system we established may be used to predict the modification pattern in individual patients with LUAD and, encouragingly, to propose novel treatments for METTL3 as a possible secondary therapeutic option for the high-risk LUAD group. Our findings demonstrated that overexpression of METTL3 significantly upregulated PD-L1 in the A549 cell line, inhibiting the anti-tumor effects of T cells. This aligns with the known function of PD-L1 in immune evasion by tumors (45). Our previous study has found that METTL3 knockdown in a breast cancer mouse model enhanced PD-1 immunotherapy efficacy by improving CD8+ T cell infiltration and reducing immunosuppressive cells, thereby promoting an anti-tumor immune environment (11). Interestingly, the addition of atezolizumab, an anti-PD-L1 antibody, partially restored the anti-tumor effects of T cells, suggesting that the immunosuppressive role of METTL3 may be mediated through PD-L1. Conversely, METTL3 knockdown did not significantly alter the anti-tumor effects of T cells, regardless of atezolizumab treatment. This suggests that role of METTL3 in immune regulation is prominent. Additionally, we examined the other key m^6^A regulatory molecules, METTL14 and WTAP (P<0.05; **Supplementary Figure 4C**). Although these molecules also showed regulatory effects on the candidate genes, the trends were not as significant as those observed with METTL3 (**Supplementary Figures 4D–G**). According to these findings, it is evident that the regulatory effect of m^6^A modifications is extensive and not solely attributable to METTL3. The interplay between various m^6^A regulatory molecules and their collective impact on gene expression and immune regulation underscores the complexity of epigenetic modifications in cancer biology. Further studies are warranted to delineate the specific roles of other m^6^A regulators in this context.

There are several limitations to this model. First, we lacked data on the immunotherapy procedures and outcomes. The clinical information for patients undergoing or scheduled for immunotherapy is not well represented in our protein databases, preventing confirmation of these results in immunotherapy-treated cohorts. Future research should integrate RNA sequences, somatic mutations, and therapeutic outcomes of LUAD patients treated with immunotherapy. The databases used in this study did not include sufficient multi-locus sampling data within single tumors, which limits the ability to account for geographic heterogeneity in intratumor immunoreactivity and may reduce model accuracy. Additionally, we were unable to perform extensive bioinformatic analyses on a larger validation cohort using RNA-sequence data. Instead, we validated our results using two large independent cohorts and an external IHC tissue microarray. Future studies should incorporate data from patients undergoing immunotherapy to further validate the model’s utility in predicting treatment outcomes.

In conclusion, we identified a seven-gene risk scoring model to distinguishing patients with LUAD with high- or low-risk. The risk score was also a factor that caused heterogeneity and complexity of individual tumor microenvironments. Further *in vitro* studies suggested that the candidate genes were regulated in an METTL3−dependent m^6^A manner. An in-depth analysis of risk patterns will improve our comprehension of the TIME and help in developing more efficient therapy approaches for patients with LUAD.

## Data Availability

Publicly available datasets were analyzed in this study. This data can be found here: TCGA, https://cancergenome.nih.gov/; GEO, GSE30219 and GSE50081.
