# Supplementary material for: Decoding the prognostic landscape of LUAD: the interplay between N6-methyladenosine modification and immune microenvironment
Source: Front Immunol. 2024 Dec 10;15:1514497. doi: 10.3389/fimmu.2024.1514497 (PMC11666524; doi:10.3389/fimmu.2024.1514497)
Supplement: Supplementary file 1 [file DataSheet1.docx]

Supplementary Material

# Supplementary Figures and Tables

**1.1 Supplementary figures**


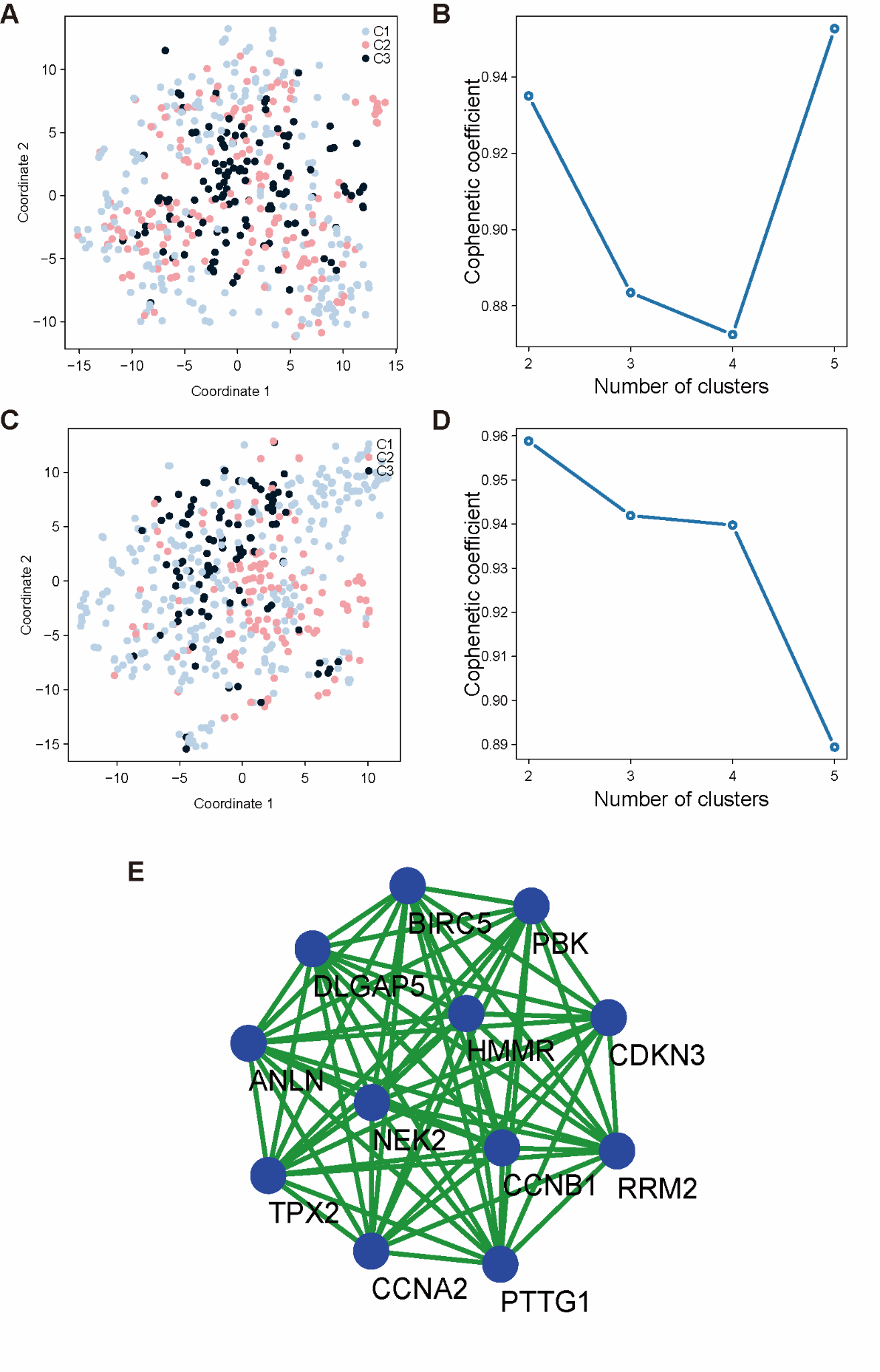


**Supplementary Figure 1.** Selection of the Immune and the m^6^A-Associated DEGs within LUAD. (A) Principal component analysis confirmed the independence among the subgroups related to immune status. (B) The optimal cluster numbers of t-SNE analysis for immune-related genes. (C) Principal component analysis confirmed the independence among the subgroups related to m^6^A status. (D) The optimal cluster numbers of t-SNE analysis for m^6^A-related genes. (E) The PPI network of the overlap genes.


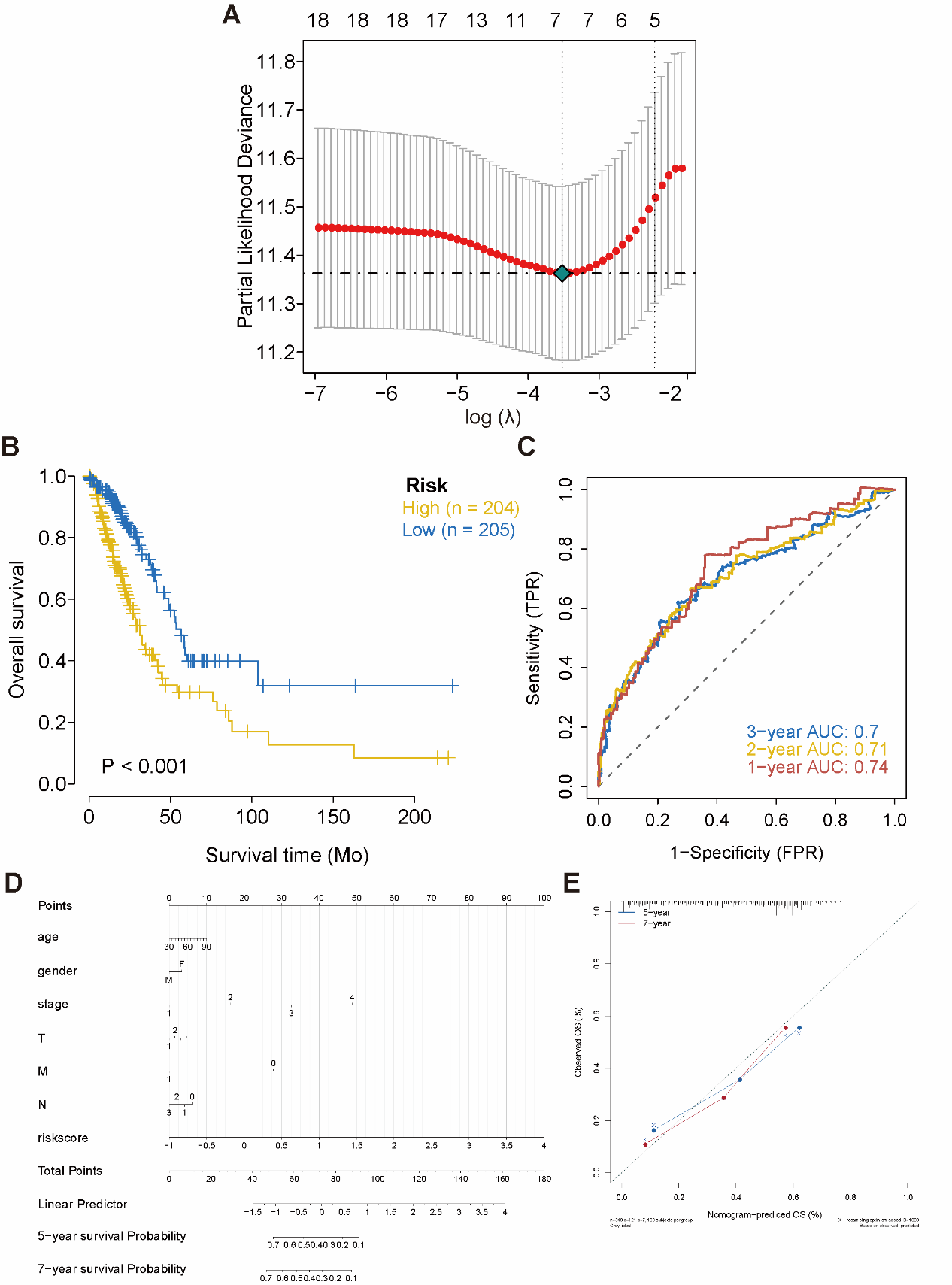


**Supplementary Figure 2.** Construction and validation of the risk score system. (A) The coefficients calculated by Least absolute1 shrinkage and selection operator (LASSO) regression. The optimal cluster numbers of t-SNE analysis for immune-related genes. (B) Internal validation of risk score system. (C) The ROC curves predicting 1/2/3-year survival by internal validation. (D) Nomogram based on clinical features. (E) Calibration plots of the nomogram for predicting the probability of OS at 5 and 7 years in the TCGA dataset.


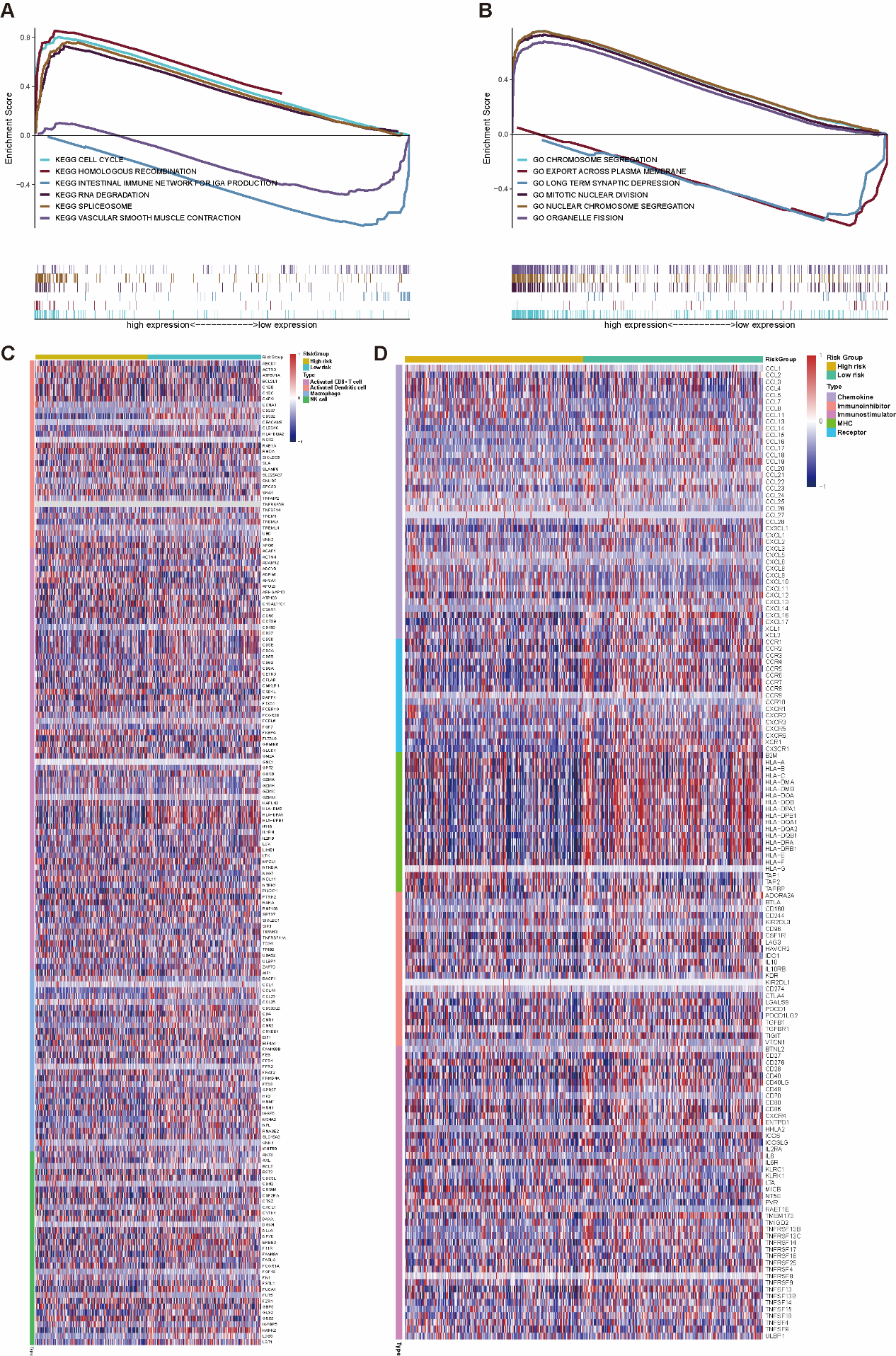


**Supplementary Figure 3.** Characteristics of the risk score system in immune subtypes. (A) GO enrichment analysis of DEGs between the low- and high-risk subgroups. (B) KEGG pathway enrichment analysis of DEGs between the low- and high-risk subgroups. (C) Heatmap of the immune cells between the low- and high-risk subgroups. (D) Heatmap of the immunomodulator and chemokines between the low- and high-risk subgroups.


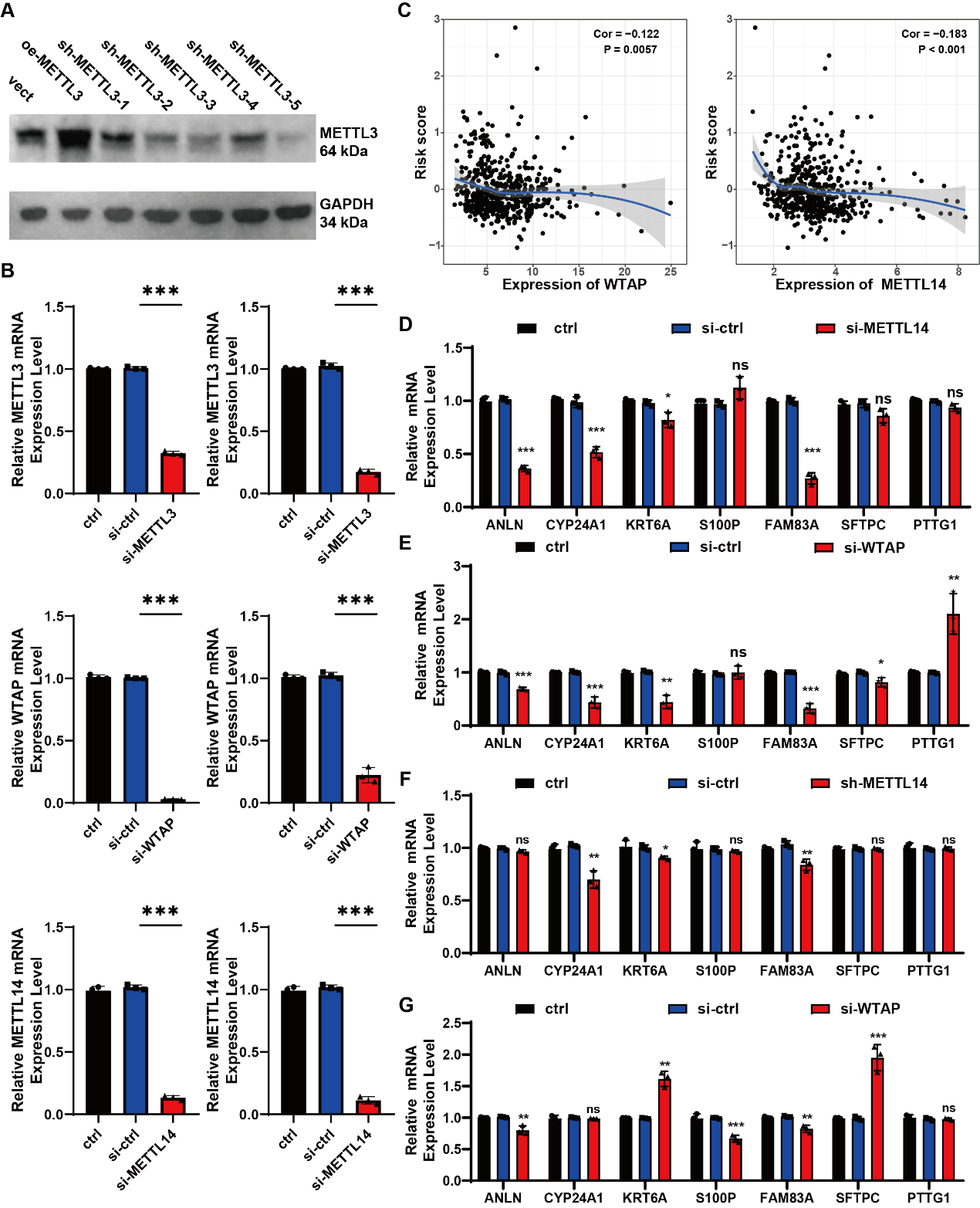


**Supplementary Figure 4.** Analysis of m^6^A Modifications and Gene Expression in NSCLC Cells. (A) Western blot showing METTL3 expression in A549 cells with overexpression (oe-METTL3) and various shRNA knockdowns (sh-METTL3-1 to sh-METTL3-5). The sh-METTL3-5 were selected for further analysis. A vector control was used as a control group to validate the overexpression efficiency. (B) Knockdown efficiency of METTL3, WTAP, and METTL14 in A549 (left) and H460 (right) cell lines. (C) Correlation between risk scores and expression levels of WTAP (top) and METTL14 (bottom). (D) The relative mRNA expression of seven candidate genes after METTL14 knocking down (METTL14-KD) in A549 cells. (E) The relative mRNA expression of seven candidate genes after WTAP knocking down (WTAP-KD) in A549 cells. (F) The relative mRNA expression of seven candidate genes after METTL14 knocking down (METTL14-KD) in H460 cells. (G) The relative mRNA expression of seven candidate genes after WTAP knocking down (WTAP-KD) in H460 cells. Data are represented as mean ± SEM of three independent experiments. Statistical significance was calculated by Student’s t-test. *P<0.05, **P<0.01, ***P<0.001, ns, non-significant.

**1.2 Supplementary tables**

**Supplementary Table 1.** The core sequences used in this study.

| Name | Sequence |
| --- | --- |
| Control siRNA^1^ | TTCTCCGAACGTGTCACGT |
| METTL3 siRNA ^1^ | CCUGCAAGUAUGUUCACUA |
| WTAP siRNA ^1^ | GGAACAGACTAAAGACAAA |
| METTL14 siRNA^1^ | GAAGACGCCTTCATCTATT |
| METTL3 ShRNA-1^2^ | ctgcaagtatgttcactatgaTTCAAGAGAtcatagtgaacatacttgcagTTTTTT |
| METTL3 ShRNA-2^2^ | gggcccaagtgcaagaattctTTCAAGAGAagaattcttgcacttgggcccTTTTTT |
| METTL3 ShRNA-3^2^ | gctgcacttcagacgaattTTCAAGAGAaattcgtctgaagtgcagcTTTTTT |
| METTL3 ShRNA-4^2^ | ggagatcctagagctattaTTCAAGAGAtaatagctctaggatctccTTTTTT |
| METTL3 ShRNA-5^2^ | gcacatcctactcttgtaaTTCAAGAGAttacaagagtaggatgtgcTTTTTT |
| GAPDH^3^ | F: GGAGCGAGATCCCTCCAAAAT |
|  | R: GGCTGTTGTCATACTTCTCATGG |
| SFTPC^3^ | F: GTCCTCATCGTCGTGGTGATTG |
|  | R: AGAAGGTGGCAGTGGTAACCAG |
| CYP24A1^3^ | F: GCTTCTCCAGAAGAATGCAGGG |
|  | R: CAGACCTTGGTGTTGAGGCTCT |
| KRT6A^3^ | F: GAGGAGATTGCTCAGAGAAGCC |
|  | R: CAATCTCCTGCTTGGTGTTGCG |
| PTTG1^3^ | F: GCTTTGGGAACTGTCAACAGAGC |
|  | R: CTGGATAGGCATCATCTGAGGC |
| S100P^3^ | F: CTCAAGGTGCTGATGGAGAAGG |
|  | R: GAACTCACTGAAGTCCACCTGG |
| FAM83A^3^ | F: ATCCAGCGCCACTGTGTACTTC |
|  | R: CCGTGAACACATCCATCAGGATG |
| ANLN^3^ | F: CAGACAGTTCCATCCAAGGGAG |
|  | R: CTTGACAACGCTCTCCAAAGCG |
| WTAP^3^ | F: GCAACAACAGCAGGAGTCTGCA |
|  | R: CTGCTGGACTTGCTTGAGGTAC |
| METTL3^3^ | F: ATCCCCAAGGCTTCAACCAG |
|  | R: GCGAGTGCCAGGAGATAGTC |
| METTL14^3^ | F: AGAGAACAAAGGAACACTGCCT |
|  | R: AATGAAGTCCCCGTCTGTGC |
| PD-L1^3^ | F: TGGCATTTGCTGAACGCATTT |
|  | R: TGCAGCCAGGTCTAATTGTTTT |

^1^ siRNA sequences

^2^ shRNA sequences for lentiviral vectors

^3^ The primers used in qRT–PCR.

**Supplementary Table 2.** Antibodies used in this study.

| Name (Cat) | Specificity | Company |
| --- | --- | --- |
| Anti-WTAP (ab195380) | IP (5 ug/per test) | Abcam |
| Anti-METTL3 (ab195352) | IP (5 ug/per test) & WB (1:1000) | Abcam |
| Anti-METTL14 (ab309096) | IP (5 ug/per test) | Abcam |
| GAPDH (D16H11) (5174) | WB (1:1000) | Cell Signaling Technology |
| Anti-SFTPC (ab90716) | IHC (1:1000) | Abcam |
| Anti-CYP24A1 (PA5-79127) | IHC (1:300) | Invitrogen |
| Anti-KRT6A (ab93279) | IHC (1:200) | Abcam |
| Anti-PTTG1 (ab79546) | IHC (1:200) | Abcam |
| Anti-S100P (ab133554) | IHC (1:1000) | Abcam |
| Anti-FAM83A (20618-1-AP) | IHC (1:200) | Proteintech |
| Anti-ANLN (ab211872) | IHC (1:200) | Abcam |

**Supplementary Table 3.** Univariate Cox analysis of immunologically and m^6^A-linked differentially expressed genes associated with overall survival.

| Gene symbol | Hazard Ratio | Z score | P value | Lower 95%CI | Upper  95%CI |
| --- | --- | --- | --- | --- | --- |
| FAM83A | 1.412148 | 5.904209 | 3.54E-09 | 1.25929 | 1.583559 |
| KRT6A | 1.320348 | 5.111004 | 3.20E-07 | 1.18688 | 1.468825 |
| ANLN | 1.37386 | 4.960907 | 7.02E-07 | 1.211836 | 1.557547 |
| HMMR | 1.259477 | 4.061083 | 4.88E-05 | 1.126773 | 1.40781 |
| DLGAP5 | 1.232751 | 3.696493 | 0.000219 | 1.103294 | 1.377398 |
| CYP24A1 | 1.230988 | 3.651354 | 0.000261 | 1.101051 | 1.376259 |
| PTTG1 | 1.271788 | 3.649419 | 0.000263 | 1.117732 | 1.447078 |
| NEK2 | 1.261932 | 3.585004 | 0.000337 | 1.111216 | 1.43309 |
| RRM2 | 1.253889 | 3.430113 | 0.000603 | 1.101828 | 1.426936 |
| CCNB1 | 1.239535 | 3.378482 | 0.000729 | 1.094351 | 1.403982 |
| CCNA2 | 1.220509 | 3.329696 | 0.000869 | 1.085426 | 1.372404 |
| S100P | 1.203971 | 3.122435 | 0.001794 | 1.071552 | 1.352755 |
| TPX2 | 1.181395 | 2.846904 | 0.004415 | 1.053306 | 1.325061 |
| CPS1 | 1.191246 | 2.800928 | 0.005096 | 1.053948 | 1.34643 |
| PBK | 1.188289 | 2.760436 | 0.005772 | 1.051298 | 1.343131 |
| CDKN3 | 1.191677 | 2.69088 | 0.007126 | 1.048786 | 1.354036 |
| BIRC5 | 1.207525 | 2.636955 | 0.008365 | 1.049604 | 1.389208 |
| SFTPC | 0.674893 | -2.60406 | 0.009213 | 0.502004 | 0.907324 |
| RACGAP1 | 1.185478 | 2.56419 | 0.010342 | 1.040908 | 1.350127 |
| SCGB3A1 | 0.192596 | -2.51818 | 0.011796 | 0.05344 | 0.694105 |
| GGT6 | 0.789082 | -2.48837 | 0.012833 | 0.654773 | 0.950942 |
| AGER | 0.68656 | -2.40068 | 0.016365 | 0.505056 | 0.933291 |
| COLCA1 | 0.63481 | -2.39771 | 0.016498 | 0.437843 | 0.920384 |
| TNS1 | 0.812948 | -2.39616 | 0.016568 | 0.686275 | 0.963004 |
| B3GNT8 | 0.795272 | -2.36699 | 0.017933 | 0.657868 | 0.961375 |
| NTS | 1.131859 | 2.352037 | 0.018671 | 1.020862 | 1.254925 |
| CPAMD8 | 0.793798 | -2.34853 | 0.018848 | 0.654655 | 0.962514 |
| MAD2L1 | 1.155802 | 2.347008 | 0.018925 | 1.024165 | 1.304358 |
| CLIC6 | 0.793344 | -2.34558 | 0.018998 | 0.65381 | 0.962657 |
| ADH1C | 0.185738 | -2.33641 | 0.01947 | 0.045248 | 0.762438 |
| ATP13A4 | 0.761305 | -2.31791 | 0.020454 | 0.604516 | 0.958759 |
| SELENBP1 | 0.805696 | -2.31026 | 0.020874 | 0.670763 | 0.967772 |
| CYP4B1 | 0.788063 | -2.30213 | 0.021328 | 0.643423 | 0.965219 |
| SCGB1A1 | 0.476039 | -2.27997 | 0.022609 | 0.251495 | 0.901062 |
| RAP1GAP | 0.821496 | -2.25515 | 0.024124 | 0.69245 | 0.974591 |
| CDC20 | 1.154993 | 2.251191 | 0.024373 | 1.018816 | 1.309373 |
| TCN1 | 1.182177 | 2.250015 | 0.024448 | 1.021809 | 1.367714 |
| CDK1 | 1.147059 | 2.234808 | 0.02543 | 1.017017 | 1.29373 |
| SFTPB | 0.81877 | -2.21572 | 0.026711 | 0.686037 | 0.977184 |
| PIGR | 0.727487 | -2.21369 | 0.02685 | 0.548893 | 0.96419 |
| CRTAC1 | 0.590367 | -2.19752 | 0.027983 | 0.368966 | 0.944621 |
| IRX2 | 0.791343 | -2.18982 | 0.028537 | 0.641798 | 0.975734 |
| SUSD2 | 0.761673 | -2.13121 | 0.033072 | 0.592975 | 0.978363 |
| COL4A3 | 0.79794 | -2.09405 | 0.036256 | 0.645977 | 0.985651 |
| CKS1B | 1.139652 | 2.062689 | 0.039142 | 1.006531 | 1.290379 |
| SLC22A3 | 0.837944 | -2.03699 | 0.041651 | 0.706861 | 0.993337 |
| DMBT1 | 0.796516 | -2.02743 | 0.042619 | 0.639259 | 0.992458 |
| CRYM | 0.816917 | -1.99782 | 0.045736 | 0.669915 | 0.996175 |
| RNASE1 | 0.826589 | -1.99378 | 0.046176 | 0.685459 | 0.996775 |
| FOLR1 | 0.837602 | -1.97416 | 0.048364 | 0.702471 | 0.998727 |
| CACNA2D2 | 0.828011 | -1.96416 | 0.049511 | 0.685879 | 0.999597 |
| MTHFD2 | 1.130247 | 1.963492 | 0.049589 | 1.00022 | 1.277176 |
| CYP2B7P | 0.704861 | -1.96047 | 0.049941 | 0.496875 | 0.999909 |
| GDF15 | 0.846672 | -1.95106 | 0.05105 | 0.71631 | 1.00076 |
| SCNN1B | 0.73357 | -1.93634 | 0.052826 | 0.536095 | 1.003788 |
| FOXA2 | 0.857031 | -1.91659 | 0.05529 | 0.731942 | 1.003498 |
| MFAP4 | 0.825758 | -1.91582 | 0.055388 | 0.678874 | 1.004421 |
| KIAA1324 | 0.83933 | -1.9046 | 0.056833 | 0.700897 | 1.005105 |
| BARX1 | 1.140724 | 1.893996 | 0.058226 | 0.995425 | 1.307232 |
| NAPSA | 0.844983 | -1.88923 | 0.058861 | 0.709507 | 1.006326 |
| AQP5 | 0.808592 | -1.88224 | 0.059804 | 0.648109 | 1.008812 |
| UBE2T | 1.134347 | 1.873828 | 0.060954 | 0.994222 | 1.29422 |
| PFN2 | 1.139095 | 1.873098 | 0.061055 | 0.993979 | 1.305398 |
| MYH11 | 0.849319 | -1.86898 | 0.061626 | 0.71563 | 1.007983 |
| GSTA1 | 0.667547 | -1.85903 | 0.063023 | 0.435947 | 1.022185 |
| NFIX | 0.830288 | -1.85796 | 0.063175 | 0.682374 | 1.010263 |
| AQP4 | 0.851032 | -1.85617 | 0.06343 | 0.717751 | 1.009061 |
| C16orf89 | 0.843815 | -1.84615 | 0.064871 | 0.704609 | 1.010524 |
| IL37 | 1.163686 | 1.845039 | 0.065032 | 0.990602 | 1.367012 |
| C7 | 0.787619 | -1.82891 | 0.067413 | 0.609821 | 1.017254 |
| KCNQ1 | 0.857529 | -1.82249 | 0.06838 | 0.72688 | 1.011661 |
| CXCL17 | 0.847424 | -1.80653 | 0.070835 | 0.708101 | 1.01416 |
| WFDC2 | 0.864066 | -1.76358 | 0.077804 | 0.734561 | 1.016403 |
| MARCO | 0.84209 | -1.76068 | 0.078293 | 0.695454 | 1.019644 |
| AZGP1 | 0.845562 | -1.72399 | 0.084709 | 0.698745 | 1.023227 |
| CCNB2 | 1.085921 | 1.695785 | 0.089927 | 0.987241 | 1.194465 |
| PMAIP1 | 1.135767 | 1.691027 | 0.090832 | 0.979957 | 1.31635 |
| ADH1B | 0.833568 | -1.62072 | 0.105078 | 0.668858 | 1.038839 |
| CYP4X1 | 0.8533 | -1.57743 | 0.114697 | 0.70064 | 1.039222 |
| ALPL | 0.857688 | -1.54689 | 0.12189 | 0.706082 | 1.041846 |
| CTSH | 0.854517 | -1.52024 | 0.128451 | 0.697738 | 1.046525 |
| CREB3L1 | 0.876736 | -1.51593 | 0.129537 | 0.739611 | 1.039284 |
| DUOX1 | 0.878537 | -1.4788 | 0.139194 | 0.739982 | 1.043036 |
| BPIFB1 | 0.881213 | -1.47737 | 0.139577 | 0.745112 | 1.042173 |
| SFTPD | 0.873052 | -1.47046 | 0.141437 | 0.72854 | 1.04623 |
| GGTLC1 | 0.839037 | -1.45318 | 0.146174 | 0.662188 | 1.063116 |
| DES | 0.857121 | -1.40984 | 0.158587 | 0.691762 | 1.062007 |
| WIF1 | 0.826247 | -1.39888 | 0.161849 | 0.632372 | 1.07956 |
| UBE2C | 1.105136 | 1.393454 | 0.163483 | 0.960173 | 1.271985 |
| INMT | 0.868076 | -1.38755 | 0.165275 | 0.710834 | 1.060101 |
| SLC44A4 | 0.891399 | -1.38322 | 0.166598 | 0.757402 | 1.049102 |
| C1orf116 | 0.887518 | -1.35653 | 0.174931 | 0.746968 | 1.054515 |
| ELN | 0.889525 | -1.3377 | 0.180995 | 0.749318 | 1.055967 |
| COL17A1 | 1.102383 | 1.316577 | 0.187981 | 0.953483 | 1.274535 |
| PSAT1 | 1.08103 | 1.297078 | 0.194604 | 0.960964 | 1.216097 |
| MMP12 | 1.095163 | 1.285844 | 0.198497 | 0.953461 | 1.257924 |
| TPPP3 | 0.890026 | -1.28567 | 0.19856 | 0.745191 | 1.063009 |
| CX3CL1 | 0.901653 | -1.27727 | 0.201507 | 0.769216 | 1.056893 |
| TMEM59L | 0.876359 | -1.24532 | 0.213014 | 0.711986 | 1.07868 |
| VSIG2 | 0.872419 | -1.24084 | 0.214664 | 0.70323 | 1.082312 |
| MMP1 | 1.082962 | 1.238749 | 0.215438 | 0.954658 | 1.228509 |
| FCGBP | 0.903651 | -1.16549 | 0.243821 | 0.762094 | 1.071502 |
| TMPRSS2 | 0.882466 | -1.11963 | 0.262871 | 0.708989 | 1.098389 |
| AGR3 | 0.91553 | -1.09959 | 0.271512 | 0.782269 | 1.071493 |
| UPK3B | 1.063506 | 1.097263 | 0.272527 | 0.952744 | 1.187145 |
| CRLF1 | 0.910289 | -1.0862 | 0.277391 | 0.768283 | 1.078542 |
| SCGB3A2 | 0.879105 | -1.08049 | 0.279923 | 0.695877 | 1.110577 |
| CLU | 0.923933 | -1.07917 | 0.280512 | 0.800271 | 1.066703 |
| LINC00342 | 0.90523 | -1.07621 | 0.281835 | 0.755109 | 1.085197 |
| CLDN2 | 0.892434 | -1.07502 | 0.282365 | 0.725215 | 1.098209 |
| TOP2A | 1.07679 | 1.060402 | 0.288962 | 0.939166 | 1.234581 |
| UCHL1 | 1.077068 | 1.05461 | 0.291604 | 0.938253 | 1.236421 |
| ROS1 | 0.905792 | -0.98379 | 0.32522 | 0.743735 | 1.103161 |
| NUF2 | 1.070685 | 0.917264 | 0.359004 | 0.925299 | 1.238915 |
| CLDN18 | 0.894376 | -0.89621 | 0.37014 | 0.700644 | 1.141676 |
| HMGB3 | 0.923224 | -0.84898 | 0.39589 | 0.76774 | 1.110195 |
| SLC22A31 | 0.937701 | -0.81485 | 0.41516 | 0.803286 | 1.094608 |
| PEBP4 | 0.936554 | -0.79862 | 0.424513 | 0.797386 | 1.100011 |
| CAPN8 | 0.948122 | -0.64819 | 0.516865 | 0.807063 | 1.113836 |
| LINC00261 | 0.935814 | -0.63073 | 0.528216 | 0.761485 | 1.150051 |
| FGB | 1.044779 | 0.626014 | 0.531306 | 0.910881 | 1.19836 |
| SULT1C2 | 1.056611 | 0.603029 | 0.546489 | 0.883459 | 1.2637 |
| AQP1 | 0.952508 | -0.53231 | 0.594514 | 0.796274 | 1.139397 |
| CXCL10 | 1.040879 | 0.499579 | 0.617371 | 0.889477 | 1.218053 |
| LGALS4 | 1.03891 | 0.493012 | 0.622004 | 0.892631 | 1.209161 |
| MMP28 | 0.95203 | -0.48546 | 0.627352 | 0.780649 | 1.161035 |
| VSIG1 | 1.045253 | 0.442724 | 0.657966 | 0.859264 | 1.271501 |
| CKS2 | 1.029399 | 0.42897 | 0.667945 | 0.901756 | 1.175109 |
| GGH | 0.972593 | -0.4131 | 0.679531 | 0.852454 | 1.109664 |
| SCTR | 1.036322 | 0.405978 | 0.684759 | 0.872347 | 1.23112 |
| PGC | 0.97258 | -0.36785 | 0.712985 | 0.838667 | 1.127875 |
| ERN2 | 0.970652 | -0.36307 | 0.716549 | 0.826477 | 1.139979 |
| ST6GALNAC1 | 0.969823 | -0.35705 | 0.721053 | 0.819679 | 1.14747 |
| HHLA2 | 0.970509 | -0.35294 | 0.724136 | 0.821871 | 1.146028 |
| MS4A15 | 0.971282 | -0.35052 | 0.725949 | 0.825251 | 1.143154 |
| TFF3 | 0.971598 | -0.34696 | 0.728624 | 0.825655 | 1.143338 |
| CTSE | 0.971181 | -0.32197 | 0.747472 | 0.812816 | 1.160401 |
| S100A8 | 0.964072 | -0.2928 | 0.769677 | 0.754641 | 1.231626 |
| SHISA3 | 0.978255 | -0.29247 | 0.769927 | 0.844244 | 1.133538 |
| GALNT5 | 1.029204 | 0.288182 | 0.773207 | 0.846209 | 1.251771 |
| APOD | 0.982148 | -0.22135 | 0.824823 | 0.837345 | 1.151992 |
| FAM83E | 1.011101 | 0.124632 | 0.900815 | 0.849949 | 1.202809 |
| S100A9 | 0.992209 | -0.08996 | 0.928321 | 0.836749 | 1.176552 |
| PAEP | 1.005957 | 0.063074 | 0.949707 | 0.836425 | 1.20985 |

**Supplementary Table 4**. The clinical feature of patients used in training set.

|  | High-risk group (n=204) | Low-risk group (n=205) | P |
| --- | --- | --- | --- |
| Survival time (mean (SD)) | 678.30 (860.76) | 827.13 (825.81) | 0.075^1^ |
| Status (Death (%)) | 87 (42.6) | 50 (24.4) | <0.001^2^ |
| Age (mean (SD)) | 63.84 (10.70) | 65.96 (9.62) | 0.035^1^ |
| Gender (MALE (%)) | 116 (56.9) | 77 (37.6) | <0.001^2^ |
| Stage (%) |  |  | <0.001^2^ |
| Stage I | 3 (1.5) | 2 (1.0) | |
| Stage IA | 34 (16.7) | 72 (35.1) | |
| Stage IB | 53 (26.0) | 58 (28.3) | |
| Stage IIA | 28 (13.7) | 15 (7.3) | |
| Stage IIB | 31 (15.2) | 27 (13.2) | |
| Stage IIIA | 34 (16.7) | 19 (9.3) | |
| Stage IIIB | 7 (3.4) | 0 (0.0) | |
| Stage IV | 12 (5.9) | 9 (4.4) | |
| unknow | 2 (1.0) | 3 (1.5) | |
| T (%) |  |  | 0.022^2^ |
| T1 | 23 (11.3) | 28 (13.7) | |
| T1a | 14 (6.9) | 26 (12.7) | |
| T1b | 15 (7.4) | 31 (15.1) | |
| T2 | 69 (33.8) | 63 (30.7) | |
| T2a | 35 (17.2) | 29 (14.1) | |
| T2b | 16 (7.8) | 6 (2.9) | |
| T3 | 23 (11.3) | 16 (7.8) | |
| T4 | 8 (3.9) | 4 (2.0) | |
| TX | 1 (0.5) | 2 (1.0) | |
| M (%) |  |  | 0.041^2^ |
| M0 | 140 (68.6) | 133 (64.9) | |
| M1 | 10 (4.9) | 3 (1.5) | |

^1^ p-values were made by Student’s t test.

^2^p-values were made by chi-square test.

**Supplementary Table 5.** The clinical feature of patients used in testing set.

|  | High-risk group (n=51) | Low-risk group (n=51) | P |
| --- | --- | --- | --- |
| Survival time (mean (SD)) | 633.41 (445.36) | 812.40 (794.09) | 0.162^1^ |
| Status (Death (%)) | 16 (31.4) | 10 (19.2) | 0.233^2^ |
| Age (mean (SD)) | 64.35 (10.06) | 66.52 (9.24) | 0.257^1^ |
| Gender (MALE (%)) | 22 (43.1) | 21 (40.4) | 0.934^2^ |
| Stage (%) |  |  | 0.238^2^ |
| Stage IA | 12 (23.5) | 17 (32.7) | |
| Stage IB | 12 (23.5) | 18 (34.6) | |
| Stage II | 1 (2.0) | 0 (0.0) | |
| Stage IIA | 5 (9.8) | 2 (3.8) | |
| Stage IIB | 4 (7.8) | 6 (11.5) | |
| Stage IIIA | 10 (19.6) | 6 (11.5) | |
| Stage IIIB | 2 (3.9) | 1 (1.9) | |
| Stage IV | 4 (7.8) | 0 (0.0) | |
| unknow | 1 (2.0) | 2 (3.8) | |
| T (%) |  |  | 0.449^2^ |
| T1 | 8 (15.7) | 10 (19.2) | |
| T1a | 3 (5.9) | 4 (7.7) | |
| T1b | 5 (9.8) | 6 (11.5) | |
| T2 | 14 (27.5) | 14 (26.9) | |
| T2a | 9 (17.6) | 11 (21.2) | |
| T2b | 5 (9.8) | 0 (0.0) | |
| T3 | 3 (5.9) | 5 (9.6) | |
| T4 | 4 (7.8) | 2 (3.8) | |
| M (%) |  |  | 0.147^2^ |
| M0 | 31 (60.8) | 35 (67.3) | |
| M1 | 4 (7.8) | 0 (0.0) | |
| MX | 15 (29.4) | 17 (32.7) | |
| unknow | 1 (2.0) | 0 (0.0) | |
| N (%) |  |  | 0.211^2^ |
| N0 | 33 (64.7) | 39 (75.0) | |
| N1 | 7 (13.7) | 9 (17.3) | |
| N2 | 10 (19.6) | 3 ( 5.8) | |
| NX | 1 (2.0) | 1 (1.9) | |
| Risk score (mean (SD)) | 0.32 (0.47) | -0.31 (0.17) | <0.001^1^ |

^1^p-values were made by Student’s t test.

^2^p-values were made by chi-square test.
